# Theoretical Model for In Vivo Induction of Chemotherapy Sensitization Using miRNA Packaged in Distinct Layered Liposomes

**DOI:** 10.3390/jfb15100298

**Published:** 2024-10-05

**Authors:** Ruxandra-Ioana Cipu, Mihai-Laurențiu Stănişteanu, Mihaela-Aurelia Andrei, Daniel Dumitru Banciu, Adela Banciu

**Affiliations:** Department of Biomaterials and Medical Devices, Faculty of Medical Engineering, National University of Science and Technology Politehnica Bucharest, 1-7 Gh. Polizu Street, 011061 Bucharest, Romania; ruxandra_ioana.cipu@stud.fim.upb.ro (R.-I.C.); mihai.stanisteanu@stud.fim.upb.ro (M.-L.S.); mihaela.andrei0911@stud.fim.upb.ro (M.-A.A.); adela.banciu@upb.ro (A.B.)

**Keywords:** chemotherapy, cancers, metabolism, treatment efficiency

## Abstract

Resistance to chemotherapy is a problem of major social and economic importance, when looking at factors like the decrease in life expectancy, the associated therapeutic costs, and a significant number of cancers that resist current chemotherapy. The development of chemotherapeutics for all theoretically possible tumor variants is an approach that requires unreasonable resources. We propose a theoretical model that serves the purpose of overcoming resistance to chemotherapeutic agents used in cancer therapy. The model describes a gene delivery system based on liposomes, which are optically guided to the tumor’s location. The main aim of the gene delivery system is inhibiting the activity of enzymes involved in drug metabolism, hence offering the opportunity to use inexpensive chemotherapeutics that are already on the market. This model will reduce the costs of chemotherapy and will assure a positive outcome for patients.

## 1. Introduction

Cancer is an illness caused by an uncontrollable proliferation of body cells, which causes the formation of a mass called a tumor. According to the World Health Organization, cancer is the second cause attributed to the worldwide death rate. Moreover, in the year 2022, a total of 1,918,030 new cancer cases and 609,360 deaths caused by cancer were projected to occur in the United States [1]. Cancer poses a significant challenge in nations regardless of their economic status. In Western countries, the incidence of numerous cancers is managed through the reduction in established risk factors, early detection methods, and advancements in treatment [2].

Tumors are typically categorized into four main groups. Firstly, they are classified broadly based on the tissue, organ, and system from which they originate. Then, they are further categorized by their specific type and graded according to World Health Organization (WHO) classifications. Tumors can be classified by their spread using the Tumor Node Metastasis (TNM) system. These classifications play a crucial role in clinical oncology, cancer research, and the education of oncologists and pathologists. Tumors are also graded according to their prognosis using the WHO grading system, which considers cytological and morphological features. Grades range numerically from one (well-differentiated) to three (poorly differentiated). Some tumors have specific grading systems tailored to their characteristics [3].

In the broader classifications based on the tissue or organ of origin, hematological cancers are differentiated from solid neoplasms [3]. The latter are further categorized into carcinomas (originating from epithelial cells, in the breasts, prostate, kidneys, skin and urinary tract [4]), sarcomas (derived from soft tissue and bones [5]), lymphomas (arising in lymphoid tissue, such as the natural killers B- and T-cells [6]), myeloma (affecting plasma cells in the bone marrow [7]), leukemia (a cancer that can arise in any type of blood cells [8]) and glioma (the most common form of central nervous system neoplasms) [9].

As previously mentioned, tumors are staged based on their extent of spread using the TNM Classification of Malignant Tumors, developed by the UICC (Union for International Cancer Control) in 1958 and recognized by the NCI (National Cancer Institute) [10] and the ACS (American College of Surgeons) [11]. According to the eighth edition of TNM Classification of Malignant Tumors [12], the TNM system categorizes the anatomical extent of disease based on three components: T (describes the size and extent of the primary tumor), N (indicates the absence or presence of regional lymph node metastasis, along with its extent), and M (specifies the absence or presence of distant metastasis). Numeric values assigned to these components (e.g., T0–T4 for primary tumor, N0–N3 for lymph nodes, and M0–M1 for distant metastasis) quantify the severity and spread of the malignant disease.

Surgery is usually the first method, when it comes to treating various forms of tumors. It aims to completely remove tumors, but sometimes vital organs are affected, making a complete removal impossible. The standard tool, the scalpel, requires cutting through tissues, leading to a recovery period that hinders regular activities. This can negatively impact mental health and resources. Less invasive surgeries like laparoscopic or laser surgery are less effective for metastatic cancer, especially in inaccessible areas.

Chemotherapy is a form of cancer therapy that uses powerful medication to target neoplastic cells and eliminate them. This treatment can remove or shrink the tumor to facilitate surgical removal [13]. Chemotherapy is mostly applicable in cases in which a systemic approach is needed, such as metastasis or inoperable tumors. Chemotherapy, which is often integrated with adjunctive treatments like radiotherapy, confronts the limitation of resistance to chemotherapy agents. This phenomenon delineates instances where cancer cells do not respond to the inhibitory effects of chemotherapeutic agents. This lack of response can be attributed to multiple exposures to the drug, or it can be inherited from heterogenous cancer cells [14]. There have been efforts to counteract this obstacle, such as the development of second and third generation drugs, alongside the exploration of combination therapies. However, these approaches are expensive and can lead to unwanted side effects caused by the interaction of the drugs. Additionally, cancer cells can adapt quickly and become resistant even to the newly found agents and drug combinations. This quick adaptation and resistance has led to the need to develop novel techniques to fight chemotherapy resistance in a prolific manner.

Another popular option is radiation therapy, which consists of using high-energy waves (x-rays or gamma rays) or particles (protons or electron beams) with the aim of destroying the cancer cells. One way in which radiotherapy affects the neoplastic cells is by damaging their DNA, which will prevent them from further division. The high-energy waves and particles can also affect the tumor in an indirect way by producing free radicals, which will damage the cancer cells. However, the major drawback of radiotherapy treatment is that, in order to deliver a lethal dose to cancerous cells, healthy tissue cannot always be spared and adverse effects after therapy sessions can severely impact the health and quality of life of the cancer patient [15]. Some of the most known side effects are the occurrence of genetic mutations, the formation of new malignancies, infertility [16], or heart disease [17]. Proton beam radiotherapy addresses the problem of radiation particles damaging healthy tissue by precisely depositing all their energy at the tumor site. This precision is due to their high velocity compared to other particles. However, accessibility to this treatment is hindered by the need for a particle accelerator, extensive infrastructure such as lengthy pipes and electromagnets, and a large gantry [18].

One kind of cancer treatment that slows or stops the growth of hormone-sensitive cancers is hormone therapy. The idea behind this treatment is that certain cancer cell types (like the ones in prostate and breast cancers) need hormones like testosterone or estrogen to proliferate and divide. For hormone therapy to be effective, the hormones must either be prevented from being produced or their receptors on cancer cells must be blocked [19].

The treatments described above have a meaningful impact when it comes down to the financial aspect, bearing in mind that radiotherapy and chemotherapy need multiple sessions and administrations in order to be fruitful. In the year 2009, the cancer expense in the EU was EUR 129 billion, while a total cost of EUR 52.03 billion was attributed to lost working days and early death [20].

Owing to the rapid advancements in medical engineering, novel treatment methodologies emerge annually.

Cancer vaccines serve as an illustrative example of novel approaches. They represent a subset of immunotherapy that aims to elicit or bolster anti-tumor immunity by leveraging the patient’s immune response, especially regarding cytotoxic CD8^+^ T cells [21].

CD8^+^ T cells adeptly discern neoplastic cells through the detection of specific antigens displayed on their surface, which can be either tumor-specific or tumor-associated. Upon antigen recognition, T cells undergo activation via intricate signaling pathways. One mechanism through which cytotoxic CD8^+^ T cells induce apoptosis is through perforin/granzyme-mediated pathways [22]. Another way to trigger apoptosis is by expressing death receptor ligands, which bind to death receptors on the surface of cancer cells. Furthermore, the production of interferon-gamma can enhance the immune response against neoplastic cells also [23].

While cancer vaccines represent a strong and promising tool for cancer treatment, several caveats warrant consideration, including tumor heterogeneity, immunosuppressive microenvironments, autoimmune reactions, and treatment personalization.

Another novel cancer option is angiogenesis inhibition. When a tumor’s radius goes over a threshold value, it cannot supply itself with nutrients and oxygen solely through a diffusion process and demands the formation of blood vessels. Once the blood vessels are formed, the risk of metastasis escalates. Angiogenesis inhibition operates through three primary mechanisms: inhibiting the release of angiogenic molecules from tumor cells, neutralizing already released angiogenic molecules, and preventing endothelial cells from responding to the stimulation of angiogenesis [24]. The main drawbacks of this technique are the development of resistance to angiogenesis inhibitors in cancer cells over time and normal tissue toxicity.

Another promising frontier is gene delivery systems. An encapsulation of miRNA into biocompatible lipidic vesicles followed by their delivery to the tumor site could modulate the expression of intracellular enzymes that metabolize chemotherapy drugs.

Current cancer treatments have various advantages and disadvantages (Table 1), which our technology tries to overcome.

## 2. Gene Delivery

### 2.1. Fundamentals of Gene Therapy

Gene therapies use either DNA or RNA. Two significant distinctions between them are their single-stranded structure and the presence of a hydroxyl group (-OH) on the carbon atom in position 2 of the ribose molecule. As a result, all RNA types (ncRNA, rRNA, tRNA, and mRNA) can adopt conformations. For example, secondary and tertiary structures have higher melting points than DNA fragments of the same length but also have a higher propensity to degrade. These structures are more complex and articulated than those that DNA assumes [31]. Among nucleic acid types, non-coding RNA (ncRNA) has garnered significant attention due to its distinct properties.

The term non-coding RNA (ncRNA) is typically used to describe RNA that does not code for proteins; however, this property does not imply that these RNAs lack information or functional roles [32]. Based on several criteria (length, shape, location, etc.), ncRNAs have been classified into microRNA (miRNA), long ncRNA (lncRNA), circular RNA (circRNA), etc. miRNAs are small RNA molecules that bind to the targeted mRNA in order to decrease protein expression. Some miRNAs have been identified as down-regulators of oncogenic genes with a suppression of tumor growth [33]. Some bacterial and eukaryotic RNAs modulate gene expression by binding directly to RNA polymerase [34]. These are examples of variables to take into consideration when developing therapeutic methods involving genetic material.

MicroRNAs (miRNAs), small interfering RNAs (siRNAs), and non-coding RNAs (ncRNAs), form a crucial category of gene therapy tools, particularly in cancer treatment. By leveraging the RNA interference (RNAi) mechanism, miRNAs and siRNAs exhibit strong efficacy in suppressing oncogenic factors, making them valuable for cancer gene therapy [35]. In our model, we preferred miRNA due to its reduced size that promotes better and easier stabilization. The main function of the miRNA molecule lies in its structure, which must be complementary to the genetic material encoding the synthesis of the targeted substance, such as a drug-metabolizing enzyme.

### 2.2. RNA Purpose and Stabilization

Despite ongoing research efforts to discover new chemotherapeutic agents, the development of resistance to these anticancer drugs proved to be a significant challenge. Researchers are investigating various causes of this resistance, yet achieving substantial progress remains elusive due to several factors that have received insufficient attention or study. Drug-metabolizing enzymes (DMEs) represent one of these factors, which have been relatively underexplored. DMEs are substances involved in the transformation of drugs and xenobiotics, and they encompass both Phase I (which often catalyze oxidation, reduction, and hydrolysis reactions) and Phase II enzymes (involved in conjugation reactions). Major Phase I enzymes include cytochrome P450s (CYPs), while prominent Phase II enzymes include glutathione-S-transferases (GSTs), UDP-glucuronosyltransferases (UGTs), and dihydropyrimidine dehydrogenases. These enzymes play critical roles in detoxifying xenobiotics and metabolizing drugs, with their activity dependent on the tissue in which they are expressed. When present in tumor tissues, these enzymes contribute to drug resistance by metabolizing drugs and rendering them inactive [36,37]. The ultimate purpose of the presented model is to ensure that the chosen genetic material enters the cells and alters the synthesis of the DME, thus reducing the drug’s degradation and enhancing the therapeutic effects.

Furthermore, additional miRNA stability is needed for better results. RNAs that are initially translated may later be temporarily translationally repressed [38]. These cellular processes can be modified to achieve a specific therapeutic outcome, which in this scenario involves preventing the degradation of miRNA molecules before they reach and bind to the target mRNA sequence. This action represents a complex time-dependent process, which can be controlled through a molecular clock designed to postpone the degradation of miRNA by stabilizing it through an RNA binding protein (RBP). A typical RBP plays a role in forming ribonucleoprotein (RNP) complexes primarily involved in gene expression. The RBP achieves this formation of RNP complexes by binding to specific sequence and/or structural motifs in RNA using modular combinations of a limited set of well-defined RNA-binding domains (RBDs) [39].

RBPs are known for their importance in cellular processes and medication-induced modifications. The RNA–RBP complex is implied in the coordination and stabilization of protein complexes, processing and maturation of mRNA (trafficking, stabilization, and silencing of mature mRNA) [40]. An RBP should be used to improve the miRNA’s stability. The main aspect to take into consideration is the structure of the transported gene. For example, a protein structured with alpha helices can mimic the molecular architecture of single-stranded ribonucleic acid (RNA), thereby facilitating stronger binding interactions. However, a focus on targeting the specific RBDs within the RBP rather than the entire molecular structure could offer a more precise approach, potentially leading to improved outcomes.

## 3. Liposome Synthesis

Liposomes are lipid-based vesicles that mimic the cell membrane and can be prepared by applying different methods. They are used pharmaceutically to deliver various substances such as drugs, genes, or other bioactive molecules [41].

Our theoretical model proposes increasing the efficiency of in vivo miRNA transport to induce chemotherapy sensitivity using anisotropically layered liposomes.

In our model, the RNA–RBP complex is encapsulated in a small liposome (L1), which is obtained from cationic lipids so that it adheres to other cells’ membrane. A larger liposome (L2) surrounds L1 and is mainly anionic thanks to PEG molecules, to be repelled by other cells. The space between the vesicles is occupied by a contrast agent that can be stimulated by laser beams in order to disintegrate the multilamellar vesicle once it reaches the tumor. Every step of the synthesis process, as well as the role of every element used, is described thoroughly in the next chapters.

Liposomal formulations utilized in gene delivery systems are predominantly composed of cationic lipids [42] owing to their ability to form complexes with DNA and RNA, which are characterized by anionic moieties. The phosphate groups in the nucleic acid backbone are the negative charge carriers of nucleic acids. Electrostatic interactions take place between the phosphate groups and the cationic lipids, thereby underpinning the structural integrity of the delivery system.

The most employed cationic lipids in liposome synthesis methodologies are DOTAP (1,2-dioleoyl-3-trimethylammonium-propane) [43], DMRIE (2,3-di(tetradecoxy)propyl-(2-hydroxyethyl)-dimethylazanium bromide) [44], and DODAC (Dioctadecyldimethylammonium chloride) [45]. These cationic lipids are obtained synthetically through an intricate series of chemical reactions and are readily procurable from different manufacturers.

The extrusion method represents an efficient liposome synthesis technique [46] that promotes the encapsulation of bioactive components [47]. This synthesis method involves forcing a lipid suspension through a series of microperforated membranes. Liposomes of defined size and homogeneity can be produced by a sequential extrusion of the usual multilamellar vesicles through microperforated membranes. The process causes no detectable degradation of the phospholipids with a good encapsulation of biomolecules [48]. It requires an extruder that holds the microperforated membrane with a syringe on both ends [49]. The liquid mixture is pushed by the syringes through the microperforated membrane, which is sequential and repeated in both directions. In the case of L1, the synthesis process starts by dissolving cationic lipids in an organic solvent such as ethanol, which will then be evaporated by rotary evaporation. The lipid film created on the round bottom wall of the container is then hydrated with an aqueous solution containing the peptide-based RNA binding protein that stabilizes the RNA. Next, the liquid that contains a lipid suspension and floating RNA protein is poured into one of the syringes from the extruder. By pushing the syringes, the liquid is forced to enter the polycarbonate membrane with a chosen diameter multiple times, forming a unilamellar liposome (L1) that encapsulates the protein-RNA composite [50].

It is important to mention that phospholipid species have different phase transition temperatures that influence encapsulation efficiency, storage stability, and in vivo stability [51]. During the process of extrusion, the lipids exhibit a fluid state that is more permeable to water, favoring the RNA encapsulation. In the case of L1, the lipids must have a sol–gel temperature that is not higher than the body’s temperature (36 degrees Celsius) to avoid degrading the RNA [50].

In case of fundamental phenotypic differences between cancerous and non-cancerous cells, such as in the case of neuroblastoma, to reduce local negative effects, L1 can use specific anchoring proteins for neoplastic cells, such as antibodies for surface proteins.

Furthermore, our gene delivery system must target as many cancer cells as possible. To ensure that happens, a liposome that encapsulates a contrast substance along with the inner liposome (L1) vesicles needs to be prepared. The technique used to create the outer liposome (L2) is the previously mentioned extrusion method. In a similar way, we need to use cationic lipids to guarantee separation between the two bilayers, as the same charge encourages rejection between the bilayers. For this synthesis step, a lipid film is created by dissolving cationic lipids in an organic solvent, which ulteriorly evaporates. The lipid film is then hydrated, and the formed lipid suspension is inserted in the extruder syringe along with a liquid that contains a contrast substance and L1s. By applying pressure, the compounds will go through a membrane multiple times, creating a liposome that encapsulates both the contrast substance, as well as the liposome that contains RNA and its alpha-helix protein. The polycarbonate membrane used to form L2 has a larger pore diameter than the one used to synthesize L1, so that the former is able to incorporate the latter, alongside the contrast substance. An appropriate choice would be a membrane with a diameter greater than 30nm (diameter example for L1), but lower or equal to 100 nm (i.e., L2), for the liposome system integration in the body’s bloodstream [52].

**Figure 1:**
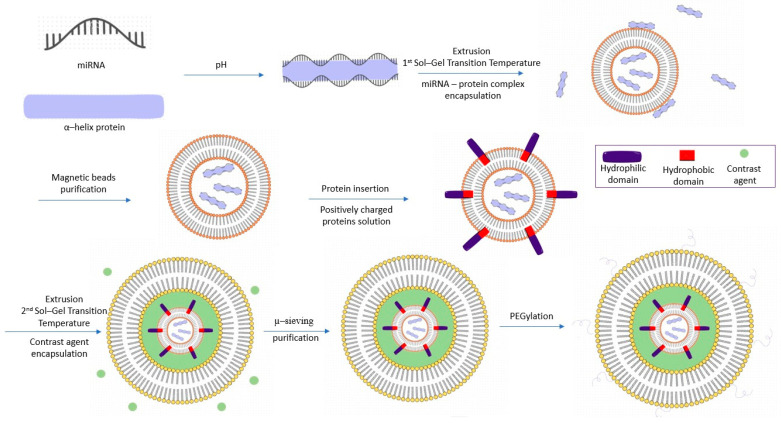
Synthesis process. The RBP–miRNA complex forms in a pH-dependent medium. Following this step, the first extrusion process encapsulates the miRNA-protein complex within L1 at its sol–gel temperature. Excess protein complexes are removed using magnetic beads for purification. Positively charged proteins with hydrophobic and hydrophilic domains are integrated into L1’s membrane. L1, along with a contrast substance, is encapsulated in L2 via the second extrusion process, which operates at a distinct sol–gel temperature. Finally, purification concludes with the µ-sieving system and PEGylation.

The stages of the synthesis of concentric liposomes (Figure 1) were chosen so that we can form liposomes with distinct concentrations of lipids, differentiated on the outer liposomes compared to the inner liposomes.

## 4. Liposome Purification

Following the extrusion process, a purification step is mandatory, to ensure that non-entrapped molecules, in our case miRNA chains and RNA-binding proteins (RBPs), will not interfere with further synthesis procedures. Given the small dimensions of L1 vesicles (0.025 µm–2.5 µm) [53], one can comprehend the need to maneuver them with care to avoid disruption of the lipid bilayer, which could lead to leakage or RNA degradation, the interest function of the genetic material then becoming lost. A purification method that can be used for the RNA-entrapped liposome is magnetic bead purification [54].

This technique is based on the use of a magnetic field, so the mechanical stress imposed on the liposomes is minimal. This way, the lipid bilayer will keep its integrity, avoiding genetic material loss. Magnetic beads are nanoparticles assembled from iron oxides, customarily Magnetite (Fe_3_O_4_) [55] with superparamagnetic properties. These nanoparticles are conventionally coated with a layer that establishes chemical bonds between the metallic bead and the molecule of interest. Since L1 is mainly composed of cationic lipids, the magnetic beads can be coated with anionic compounds, such as alginate [56], lipids [57], polysaccharides (heparin) [58], hence achieving liposome-bead binding by electrostatic interaction. The coated magnetic beads are added to the tube that contains the suspended liposomes in a buffer solution, which maintains the stability and functionality of the lipid vesicles. Then, with the aid of a pipette, the volume of the flask is aspired and dispensed back in the tube slowly several times to achieve a thorough attachment of the beads on the surface of the liposomes.

The next step is directing the now bead-binded L1 vesicles to one side of the container to wash away the unattached molecules existent in the suspension (RNA, lipids). This step is achieved with the help of a magnet, which attracts the metallic beads, given their paramagnetic properties. While the liposomes are kept in place by the magnetic field, the buffer solution is extracted with a pipette. Afterwards, the tube and its contents are washed with a concentration of 70% ethanol twice. Ethanol dislodges poorly binded lipids and leads to RNA precipitation [59]; therefore, these can be easily discarded. The magnetic field’s action is now interrupted. With the intention of disrupting the interaction between the magnetic beads and the liposomes, a solution that will disrupt the pH is added. When a pH disruptive solution is added, the surface charge of the beads and the lipid vesicles changes due to protonation; therefore, the electrostatic interactions are weakened. With a magnet, the now free beads are collected on the tube’s wall, while the liposome suspension is carefully collected in a new container.

Following the second extrusion process, the collected suspension contains miRNA encapsulated liposomes, miRNA liposome and contrast media liposomes, and residual contrast media, lipids, RNA, and proteins. Now acknowledging this step, a subsequent purification step is mandatory.

For the purpose of first separating the liposomes from the other molecules, the magnetic bead technique will be used as described earlier on. Afterwards, with a focus on separating the small miRNA liposomes from the larger miRNA liposome and contrast media liposomes, a microfluidic filtration system will be used.

Yoon et al. describe a µ-sieving system that prevents the typical membrane clogging of usual microfluidic filters [60]. The suspension containing the liposomes is inserted into the system and afterwards, low-frequency oscillations are imprinted on the fluid, while the filter remains still. The oscillatory movements prevent the lipid vesicles from aggregating on one side of the sieve. After the filtration is over, the larger liposomes can be collected and continue the manufacturing process.

## 5. Liposome PEGylation

Considering that mammalian cells pose a negative surface charge at a physiological pH, further functionalization of the liposomal system is imperative. To address this requirement, a PEGylation process represents a prolific method to meet the scope.

Polyethylene glycol (PEG) stands as an amphiphilic polymer derived from oxyethylene monomers, characterized by the chemical formula HOCH_2_(CH_2_OCH_2_)_n_CH_2_OH, where “n” represents the number of monomeric groups. PEG boasts a diverse assortment of applications, spanning from targeted drug delivery systems to wound healing, owing to its biocompatibility and diminished immunogenicity.

The purpose of PEGylating L2 vesicles and the contrast media is the ability to manipulate its charge. Although PEG itself is a neutral molecule, it can acquire a negative charge in response to surface modifications [61]. Giving the liposomal system a negative net charge would thus ensure its aversion to body cells. Imparting a negative net charge to L2 would ensure that the lipid vesicle does not adhere to tissue that is not of interest. PEGylation facilitates prolonged circulation time and controlled release.

After the second purification step, the liposomes are redispersed in a buffer solution to which PEGylated lipids are added. Subsequent incubation allows for the integration of PEGylated lipids into the phospholipid bilayer [62]. The incubation is conducted at a temperature situated slightly above the transition temperature of the lipids, ensuring a fluid-like state of the bilayer, therefore an easier incorporation of PEGylated lipids. According to D’souza and Shegokar, PEG 100 to PEG 700 exist in a liquid state at room temperature, thereby aiding their insertion into the liposome’s membrane, while PEG 1000 to PEG 2000 are soft solids [63]. Alternatively, amphiphilic proteins can serve as substitutes for PEG.

The resultant liposomal system can be purified using the magnetic bead technique previously discussed, culminating in the final refinement of the product.

## 6. Laser Usage

For the liposomes to enter a more liquid phase and lose the proteins in their membranes, they must reach surroundings with a higher temperature. Therefore, the tumor needs to be heated above body temperature (>36 °C), but not exceeding 40 °C with the purpose of keeping the patient safe and avoid physiological upregulation [64].

To keep the healthy tissue safe, especially the skin, which can become slightly irritated, multiple laser beams will reach the patient from different angles. The waves of laser will intersect in the tumor, heating it. In order to protect the patient’s healthy tissue, we must establish some parameters.

Light tissue contact involves two fundamental concepts: scattering and absorption. For the intended therapeutic outcome, absorption is essential. If there is greater absorption and less scatter, less energy will be needed to heat the target to the correct temperature and produce the desired effect. Furthermore, the penetration is more superficial the higher the absorption. The wavelengths near the Infrared (200–600 nm) have a more superficial absorption and the ones exceeding 650 nm penetrate deeper into the tissue. It is essential to also take into account the tumor’s depth into the body and simultaneously the recommended wavelengths [65].

According to the “Environment, Health and Safety” governmental website, the degree of harmful health consequences linked to ultraviolet radiation exposure is determined by the exposure duration and wavelength. Actinic UV, or wavelengths below 315 nm, were found to have the most detrimental impacts on health. Thus, the wavelength of these lasers should have a wavelength of over 400 nm in order to protect the patient, but not exceeding 1200 nm [66].

When a number of photons are directed to a delimited area, a temperature rise takes place at the target as the energy creates its thermal effect to the target tissue. In this case, having more than one laser directed to the tumor will create the power density we need to increase the temperature without heating up the skin, which would be inconvenient for the patient.

## 7. In Vivo Mechanism of Action

For this treatment to work at maximum efficiency, the gene delivery system should be paired with chemotherapy. The plan is to first weaken the neoplastic cells with this gene inhibitor system, ulteriorly administering chemotherapy or other medication such as antibiotics.

The objective of this gene delivery system is to degrade the cancer cells, consequently enabling drugs or antibiotics to eradicate the tumor with greater accuracy. The treatment’s capacity for localization stems from the liposome’s thermosensitivity, which causes its fluidity to alter in response to localized heat within the tumor, hence minimizing impact on healthy cells.

This system consists of a liposome that encapsulates RNA binded with a protein that stabilizes the gene. To further enhance the localization of the treatment, L1 has proteins in its membrane that have a hydrophobic part located in the lipid membrane and a hydrophilic part on the surface. These proteins have the ability to change polarity at a different pH with the purpose of distinguishing tumor cells with an acidic pH that varies around 6.5, from the healthy ones that have a normal pH (=7.4) [67]. Thus, the protein coating influences liposomes to change their negative charge into a positive one when in proximity of the acidic pH of the neoplastic cells [68]. By virtue of this specific characteristic, L1 exclusively infiltrates the cancerous cells while entirely passing the healthy ones. Aggregation or fusion with other liposomes is avoided due to the fact that vesicles with the same charge will not adhere to each other. Further adherence to healthy cells will not take place because of the normal pH (=7.4) that left the protein coating unchanged, thus negative and averted by the cell’s membrane that is also negatively charged [69]. The mechanism employs two steps of localization, one reliant on temperature and the second dependent on the pH. L2 changes fluidity and deliberates L1 only in proximity of the tumor due to the fact that it is heated. And second, there is the ability of proteins inserted on L1’s surface to change polarity in order to avoid adhering to healthy cells.

Gene therapy success relies on efficient and safe transfection vectors, with viral and non-viral types being commonly used. Non-viral vectors are preferred due to potential immunogenic consequences. The best transfecting vectors among the many synthetic carriers used in gene therapy are cationic liposomes. Cationic transfection lipids have benefits such as rapid manufacturing, convenient handling and preparation, capability of delivering large lipid-DNA complexes, and a low immunogenic response [70]. Enclosing a gene in liposomal vesicles prevents RNA from degrading during storage or the systemic circulation of the gene encoding a therapeutic protein, and it also enables the condensation of RNA plasmid into a highly ordered structure. Some of the structural elements in these lipids that have a significant impact on transfection efficiency are present in this delivery system. Among these are positively charged head groups that interact with a negatively charged RNA molecule, a linker group that controls the lipid’s chemical stability and biodegradability, and a hydrophobic region that anchors the cationic lipid to the bilayer [71]. Adherence followed by endocytosis happens because of two main reasons. First, the tumor is heated at a temperature slightly higher than the body’s temperature (max 40 degrees Celsius). This allows the liposome to enter a more liquid state in the vicinity of the tumor, optimizing protein movement, preceded by full protein departure from the lipid membrane [46]. Hence, the coating that brought the liposome near the tumor via electric charge exits the elasticized membrane, leaving the cationic lipid to accentuate liposome adherence to the negatively charged cell membrane.

Endocytosis is a process where eukaryotic cells absorb macromolecules and particles from the surrounding media. A region of the plasma membrane surrounds the material bound to it to be internalized during endocytosis. The cell membrane will fuse with the bilayer of the liposome, which then buds off inside the cell to form a vesicle that contains the material that has been ingested. In this case, our absorbed cell is the RNA-carrying liposome [72]. As soon as the mRNA is released in the cytoplasm, the cell’s machinery transcribes and translates the genetic material, leading to the expression of the desired gene. However, this expression is not merely a passive occurrence; it initiates a series of effects within the cell. After the RNA molecule enters the nucleus of the cell, the genetic blueprint is accurately duplicated. Subsequently, the just-created mRNA undergoes processing procedures to guarantee its stability and usefulness. After reaching maturity, the mRNA is transported to the cytoplasm, where the process of translation occurs. Ribosomes in this context are responsible for interpreting the mRNA sequence and producing a particular protein based on the given instructions. This protein, synthesized using the mRNA’s genetic code, is specifically designed to suppress the enzymatic activity in the tumor cells [73]. The proposed therapeutic miRNA induces a degradation of mRNA and subsequently inhibits protein production. Secondary, a decrease in the enzymes that metabolize the targeted chemotherapeutics can be induced, leading to a specific sensitivity.

This inhibition modifies the cellular reaction to chemotherapy, making the tumor cells more vulnerable to the impact of the treatment.

**Figure 2:**
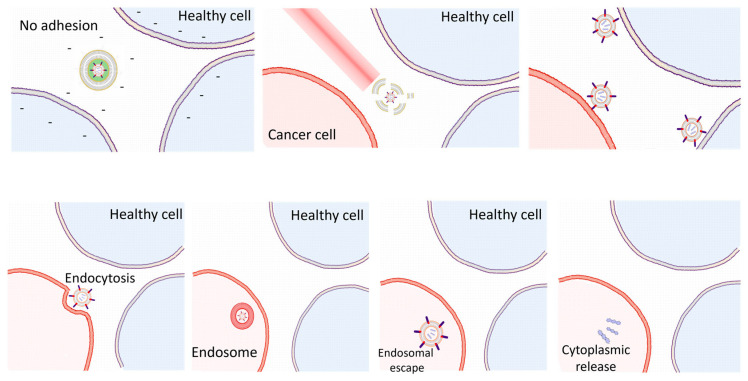
Mechanism of action. The gene delivery system features liposome complexes with a negative coating, attributed to PEG insertion in L2, preventing adherence to cells. Upon laser-induced heating of the tumor, liposomes near the site undergo a sol–gel temperature shift, releasing L1. The inclusion of a contrast agent enhances localization accuracy. Remaining L1 vesicles adjust their charge in response to pH, acquiring a positive charge near the tumor, aiding in endosome formation with neoplastic cell membranes. Subsequent endosomal escape releases the protein-miRNA complex into the cytoplasm, facilitating gene transportation.

The action steps of the liposomes (Figure 2) are in the mirror of the production steps that raised the needs of such a construction.

## 8. Discussion and Limitations

The proposed method allows the targeted delivery of miRNA to block the synthesis of enzymes capable of degrading a cheap chemotherapeutic, thus allowing chemotherapy to be more guided. Therapeutic staging, with a first step of induction of chemotherapy sensitivity and a second step of chemotherapy destruction of cancer cells, allows the use of an additional mRNA encoding fluorescent proteins, for a high-performance external control to evaluate the stages of optically guided targeted delivery of RNA.

In order to achieve this objective, liposome extrusion stages can be used at different temperatures, to make sequential liposomes on concentric layers, depending on the sol–gel temperature of the composite lipids. Thus, the external liposome (L2) ensures repellency against other cells and transports together with internal liposomes and an ultrasound-enhanced substance that allows for an optically guided rupture. The inner liposomes (L1) have the membrane polarity chosen to adhere very quickly to all the surrounding cells and thus deliver the target material as close as possible to the rupture of the outer shell liposomes.

This method can thus use the same chemotherapy for a large genetic variety of tumors, reducing the pressure on the medical systems and increasing the population’s access to efficient treatment.

The theoretical model proposes a laser targeting of areas that are to be destroyed with chemotherapy. The two-stage approach (initially laser stimulation of the transfection and then chemotherapy treatment) brings the advantages of external targeting radiotherapy, but with a decrease in the cone effect of radiotherapy, as well as the effectiveness of chemotherapy, with an increase in the effectiveness of available chemotherapeutics (at reduced concentrations), without significant general toxic effects.

To facilitate a more in-depth discussion, a SWOT analysis was conducted, encompassing an evaluation of strengths, weaknesses, opportunities, and threats. The proposed method’s strengths lie in its ability to enable a targeted delivery of miRNA, effectively blocking the synthesis of enzymes responsible for degrading affordable chemotherapeutics. This targeted approach improves the accuracy and effectiveness of chemotherapy, making the treatment process more guided. Furthermore, the therapeutic stage technique, which involves initiating chemotherapy sensitivity and subsequently targeting the elimination of cancer cells, provides a comprehensive treatment approach. The technology can utilize mRNA-encoding fluorescent proteins to achieve precise external control, facilitating the real-time assessment of treatment stages. Nevertheless, this technique has certain limitations, including vulnerability to quick removal from the bloodstream and an inability to be used for blood malignancies. The intrinsic toxicity of cationic lipids creates a fundamental constraint in gene delivery techniques. Although cationic and ionizable cationic lipids are widely used, their negative consequences may limit their application [74]. Furthermore, it is imperative to cease treatment during periods of fever to prevent liposomal leakage and ensure the continued effectiveness of the treatment.

Although there are difficulties, there are also many opportunities available. One such opportunity is the ability to overcome chemotherapy resistance and lower treatment expenses by utilizing current chemotherapeutic medicines. Due to its prolonged duration of action, this therapeutic approach can be combined with any chemotherapy agent, such as methotrexate, which has a low price and a high response rate [75]. However, the significance of meticulous risk control is highlighted by the presence of potential dangers such as contrast-induced nephropathy and laser-related side effects.

Overall, the proposed method is a promising breakthrough in cancer treatment, providing improved accuracy, effectiveness, and availability to a diverse group of patients.

In order to evaluate the functionality of the technology for a particular case, cotransfection can be used, with the addition of an mRNA for a fluorescent protein alongside the miRNA, with the evaluation by in vivo fluorescence microscopy. Sometimes, due to the distances to the optical components, this approach is not efficient. In this case, it is necessary to use some indirect evaluations, such as the evaluation of cell lysis through the evaluation of intracellular markers released in the blood flow.

The SWOT analysis of the proposed technology is summarized (Table 2) in order to highlight the proposed advantages for a quick testing and quick acceptance by health services.

## Figures and Tables

**Table 1:** Various treatment options for cancer, description, and main drawbacks [25,26,27,28,29,30].

Treatment	Description	Drawbacks
Surgery	Resection of malignant tissues from the body utilizing specialized surgical instruments, resulting in tumor eradication and prevention from neoplastic cell dissemination to distant anatomical sites.	Prolonged recovery duration; risk of complications; cosmetic effects; incomplete removal leading to risk of disease recurrence.
Chemotherapy	Utilization of pharmacological agents that impede proliferation and functional activities of neoplastic cells.	Non-selective cytotoxicity; development of resistance; psychological impact; immune system suppression.
Radiation therapy	DNA integrity disruption and free-radical synthesis through exposure to high-energy waves and/or particles.	Healthy tissue damage; secondary cancer development through ionizing radiation exposure; limited penetration.
Hormone therapy	Manipulation or blockage of hormonal receptors.	Osteoporosis and bone loss; ovarian function suppression; potential for disease recurrence.
Cancer vaccines	Initiation of an immunological cascade within the host organism meant to discern and assail neoplastic cellular entities.	Tumor heterogeneity; immunosuppressive tumor microenvironment; limited efficacy.
Angiogenesis inhibition	Deliberate thwarting of neovascularization, the main instrument in furnishing tumors with nutrients crucial for sustenance and proliferation.	Resistance development; healthy tissue toxicity; delayed onset of action.

**Table 2:** SWOT analysis of the presented method [76,77].

Strengths	Weaknesses	Opportunities	Threats
Biocompatible, nonimmunogenic, non-toxic	Susceptible to fast clearance from the bloodstream	Counteracting chemotherapy resistance	Contrast-induced nephropathy in patients with renal dysfunctions
Targeted delivery	Not applicable to blood cancers	Reducing the costs of chemotherapy by eliminating the need to develop novel drugs	Scars, pigmentary disorders, and hair loss as side effects to laser usage
L2s do not aggregate due to their similar surface charge; therefore, preventing the formation of clots	The treatment must be put on hold if the patient is dealing with fever in order to prevent the liposomes from changing their fluidity and leak the encapsulated contents		Adverse effects caused by the toxicity of cationic lipids
Attaches to target cells through electromagnetic interactions, hence healthy cells are not affected			
Cost effectiveness			
